# Kidney function as an underestimated factor for reduced health related quality of life in patients with Fabry disease

**DOI:** 10.1186/1471-2369-15-188

**Published:** 2014-11-29

**Authors:** Martin Wagner, Johannes Krämer, Elisabeth Blohm, Dorothee Vergho, Frank Weidemann, Frank Breunig, Christoph Wanner

**Affiliations:** Department of Medicine I, Division of Nephrology, University Hospital Würzburg, Oberdürrbacherstr. 6, Würzburg, 97080 Germany; Institute of Clinical Epidemiology and Biometry, University of Würzburg, Oberdürrbacherstr. 6, Würzburg, 97080 Germany; Comprehensive Heart Failure Center, University of Würzburg, Oberdürrbacherstr. 6, Würzburg, 97080 Germany; Department of Medicine I, Division of Cardiology, University Hospital Würzburg, Oberdürrbacherstr. 6, Würzburg, 97080 Germany; Department of Anaesthesia and Critical Care, Zentrum Innere Medizin, University Hospital Würzburg, Oberdürrbacherstr. 6, Würzburg, 97080 Germany

**Keywords:** Quality of life, SF-36, Chronic kidney disease, Fabry disease

## Abstract

**Background:**

Impairments of health related quality of life (HRQoL) are frequently observed in Fabry disease (FD) and are known to be related to neuropathic pain and cardiovascular events. This study aimed to explore the role of chronic kidney disease (CKD) in a large cohort of patients with FD.

**Methods:**

In 96 patients (53% female; age 40 ± 12 yrs) with genetically proven FD, HRQoL was assessed by the Medical Outcomes Study (SF-36) questionnaire. All patients were naïve to enzyme replacement therapy. Three categories for kidney dysfunction were chosen, eGFR ≥/<60 ml/min/1.73 m^2^ or need of renal replacement therapy (RRT). Minor (e.g. arrhythmia, angina pectoris, etc.) and major (e.g. myocardial infarction, coronary artery bypass, stroke or implantable cardioverter-defibrillator) vascular events as well as pain and pain therapy were considered in linear regression analyses with the dimensions of HRQoL.

**Results:**

Ten patients (10%) had impaired kidney function and a further nine were on RRT (9.4%). Kidney function and pain emerged as the main factors associated with lower scores on the SF 36, in particular on physical components (PCS beta-coefficients for CKD −6.2, for RRT −11.8, for pain −9.1, p < 0.05, respectively), while controlling for gender, vascular event and pain-therapy. Relationships were found for mental aspects of HRQoL. Age and history of vascular events were not related to HRQoL.

**Conclusion:**

Cardiovascular events and pain are important factors related to HRQoL, social functioning and depression. Our study highlights impaired chronic kidney disease, in particular after initiation of RRT, as a strong determinant of reduced HRQoL in FD.

**Electronic supplementary material:**

The online version of this article (doi:10.1186/1471-2369-15-188) contains supplementary material, which is available to authorized users.

## Background

Fabry disease (FD) is a rare, X-linked lysosomal storage disorder, which results from deficient activity of the enzyme α-galactosidase (α-Gal A) and progressive lysosomal deposition of globotriaosylceramide (GL-3) in several tissues and cells throughout the body [1]. In classically affected males, cellular dysfunction leads to progressive organ failure [2]. In heterozygous women onset of symptoms is frequently delayed, but can show a broad variety of disease manifestation [3, 4]. Early symptoms of the disease include neuropathic pain (acroparaesthesia), triggered by fever or physical exertion, hypohidrosis and heat intolerance. Later stages are characterized by severe organ involvement such as chronic kidney disease (CKD) and end stage renal disease (ESRD), left ventricular hypertrophy and heart failure as well as cerebrovascular complications. Further classical features are angiokeratoma, corneal opacities, tinnitus, hearing loss and vertigo [2].

Management of the disease was primarily symptomatic until enzyme replacement therapy (ERT) became available for clinical use. Several clinical trials demonstrated the efficacy of ERT to prevent or slow serious organ dysfunction [5, 6]. However, longitudinal open label studies in patients with more progressed disease had a less favourable outcome. Impaired kidney function and overt proteinuria at the time of institution of ERT emerged as an important prognostic factor for the course of the disease [7].

Health Related Quality of Life (HRQoL) is a multidimensional construct referring to patients’ perceptions of the impact of disease and treatment on their physical, psychological and social function and well being. HRQoL questions about perceived physical and mental health and function have become an important component of health surveillance and are generally considered valid indicators of service needs and intervention outcomes. In Fabry disease, patients suffer not only from the debilitating physical symptoms, but also from a compromised HRQoL, which is reported for both, males and females [8, 9] Possible underlying reasons are numerous, ranging from disease related pain to vital organ dysfunction.

The purpose of the current study was to characterize HRQoL as assessed by the SF-36® questionnaire in a large cohort of patients with Fabry disease. We aimed to identify factors associated with the various dimensions of physical and mental functioning, with a focus on kidney function.

## Methods

### Study participants

Since June 2001, an increasing number of patients with genetically proven Fabry disease from all over the country are overseen at our hospital. In regular visits, physical examination and detailed evaluation of medical history and medications are performed by a trained nurse and a physician. Furthermore, patients are enrolled in an international observational cohort study if they provided written informed consent [10]. The local Ethics Committee of the University of Würzburg approved the study. For the current analyses, patients who had their first visit until August 2009 were included, if they were older than 16 years with available data on HRQoL within three months before or after the first visit. We analyzed data from the first visit at our center, regardless of time since the diagnosis of Fabry disease or whether or not they were receiving ERT at the time of enrollment.

### Assessment of HRQoL

HRQoL was assessed by the Medical Outcomes Study SF-36 questionnaire [11] at baseline and was repeated after six months [2]. The SF-36 is a validated instrument to describe HRQoL in a broad spectrum of diseases and is widely used in clinical practice and clinical research [12, 13]. Patients respond to questions about activities of daily life and physical and social well being, resulting in two summary scales (physical component summary [PCS] and mental component summary [MCS]) and eight scales describing various physical and mental aspects (physical and social functioning, physical and emotional role, bodily pain, general and mental health, vitality). Each dimension is displayed on a scale ranging from score 0 to 100, representing impaired vs. improved health [14].

### Definitions

Glomerular filtration rate (eGFR) was estimated according to the simplified Modification of Diet in Renal Disease formula [15]. Stages of CKD were classified as eGFR ≥ 60 ml/min/1.73 m^2^, eGFR < 60 ml/min/1.73 m^2^ and renal replacement therapy (RRT), i.e., dialysis or kidney transplantation (KTx). History of arrhythmia, angina pectoris, significant cardiac procedure without coronary stent implantation, or transient ischemic attack (TIA) was considered as minor vascular event, whereas history of myocardial infarction, coronary stent implantation, coronary artery bypass, stroke or implantable cardioverter-defibrillator (ICD) were described as major vascular event. Pain was assessed by using the visual analog scale (VAS), ranging from no pain (score 0) to maximal pain (score 10). Patient’s pain therapy (i.e. frequent use of analgetics) was not altered.

### Statistical methods

Analyses were performed using SAS 9.1 (SAS Institute, Cary, NC). Characteristics of study participants and the different scales of HRQoL were compared across CKD stages using ANOVA, Kruskal-Wallis test, χ^2^- test, and Fisher’s exact test, as appropriate. Factors associated with the various dimensions of HRQoL were examined using linear regression analysis. To assure a linear relationship with the dependent variable, the logarithmic form was used for “time since Fabry diagnosis”. In multivariate analyses, the following variables were included in the model; age, gender, kidney function, vascular event, pain and pain-therapy. We investigated the relationship between pain and HRQoL according to their interaction with pain-therapy. Notably, the variable “enzyme replacement therapy, initiated within three months following the first visit” was not considered as a risk factor for HRQoL since impaired physical and mental status of the patient represents the cause of initiating ERT; i.e., the variable can be seen as a measure of “sickness” and does not describe the effect of ERT.

The robustness of the results was further evaluated by (1) including only four variables in the multivariate model, to minimize the risk of observing potential associations by chance due to a large number of candidate variables (“overfitting”). Thus, the least significant variable was ignored; (2) including patients in whom data of the SF-36 was available within nine months prior or following the first visit (three additional patients); (3) considering kidney transplant recipients (n = 2) not in the category of RRT, but according to eGFR within the respective CKD category; (4) not differentiating between minor and major CV events, but including CV events as a single category.

## Results

### Study population

Ninety-six patients older than 16 years of fifty-two families were treated at our center between June 2001 and August 2009. The mean age of the cohort was 40 yrs, 47% were male, and the diagnosis of Fabry disease was made a median time of nine months prior to the first visit. All patients were naïve to ERT. Characteristics of study participants stratified by kidney function are displayed in Table 1. Most of the patients, in particular most women, had preserved kidney function, whereas patients with impaired kidney function were more likely to be male. All patients receiving RRT were of male gender (n = 9), of whom two underwent kidney transplantation and seven received dialysis. Minor vascular events were also more common in patients with CKD, as was a history of major vascular events. Furthermore, patients with impaired kidney function, in particular those receiving RRT, were more likely to be initiated on ERT within three months following the study visit. Written informed consent was obtained for participation and publication of clinical and individual data.

**Table 1:** **Characteristics of study participants, stratified by kidney function**

	eGFR ≥60 ml/min/1.73 m ^2^ n = 77	eGFR <60 ml/min/1.73 m ^2^ n = 10	RRT (dialysis or KTx) n = 9	p-value
Age, yrs	38.8 ± 13.1	53.0 ± 11.3	37.0 ± 8.6	0.004
Male gender	39.1 %	60.0 %	100.0 %	0.002
Diagnosis Fabry disease, months	9 (6; 14)	12 (8; 22)	14 (7; 94)	0.31
Enzyme replacement therapy^a^	37.7 %	60.0 %	88.9 %	0.01
BMI, kg/m^2^	22.4 (20.8; 24.9)	23.6 (20.9; 30.8)	21.9 (19.9; 24.4)	0.36
Systolic blood pressure, mmHg	118 ± 15	133 ± 21	131 ± 25	0.01
Diastolic blood pressure, mmHg	75 ± 10	81 ± 14	81 ± 9	0.07
eGFR, ml/min/1.73 m^2^	96 (83; 110)	37 (25; 45)	dialysis 7 (5; 13) KTx 37 (19; 54)	<0.001
Vascular event				0.05
Minor^b^	13.2 %	40.0 %	22.2 %	
Major^c^	4.0 %	10.0 %	0 %	
Pain	44.3 %	77.8 %	71.4 %	0.08
Pain therapy	18.8 %	60.0 %	62.5 %	0.002
VAS^d^	2 (1; 3)	2 (0; 3)	2 (2; 3)	0.46

Data are means (± SD), medians (IQR) or proportions; comparison across CKD stages using ANOVA, Kruskal-Wallis test, χ^2^- test or Fisher’s exact test, as appropriate; *abbreviations:* *eGFR* estimated glomerular filtration rate as assessed by the abbreviated modification of diet in renal disease (MDRD) formula, *RRT* renal replacement therapy (dialysis, or kidney transplantation [KTx]), *BMI* body mass index; VAS, visual analog scale.

^a^Initiation of enzyme replacement therapy within the first three months following the first visit.

^b^History of transient ischemic attack (TIA), angina pectoris, significant cardiac procedure without coronary stent, arrhythmia.

^c^History of stroke, myocardial infarction, coronary stent, coronary bypass surgery, ICD-implantation.

^d^Median pain according to the visual analog scale during the three months preceding the first visit in patients reporting pain.

### Health related quality of life

Data on HRQoL were missing in six patients, and in three additional patients the SF-36 was not completed within three months following the first visit, thus leaving eighty-six patients for further analyses (Table 2). Of note, all patients with missing HRQoL had preserved kidney function. Chronic kidney disease was generally associated with reduced HRQoL. More specifically, lower scores on dimensions of mental health, as represented by social functioning, role emotional, mental health, and the MCS, were observed in patients dependent on RRT. In contrast, physical aspects, such as physical functioning, role physical, bodily pain, and the PCS, were affected even in patients with moderately impaired kidney function, i.e., eGFR < 60 ml/min.

**Table 2:** **Health related quality of life (SF-36), stratified by kidney function**

	eGFR >60 ml/min/1.73 m ^2^ n = 66	eGFR <60 ml/min/1.73 m ^2^ n = 10	RRT (dialysis or KTx) n = 9	p-value
Physical functioning	85 (65; 100)	52.5 (25; 75)	50 (30; 55)	<0.001
Role physical	100 (25; 100)	37.5 (0; 100)	0 (0; 37.5)	0.004
Bodily pain	68 (51; 100)	41 (41; 64)	22 (12; 51)	0.004
General health	57 (46; 79.5)	45 (35; 52)	25 (17; 40)	<0.001
Vitality	52.5 (32.5; 65)	40 (30; 50)	30 (5; 30)	0.01
Social functioning	87.5 (62.5; 100)	75 (50; 100)	25 (25; 50)	<0.001
Role emotional	100 (66.7; 100)	100 (0; 100)	33.3 (0; 33.3)	0.008
Mental health	66 (52; 80)	64 (52; 76)	44 (28; 60)	0.08
Physical component summary	49.8 (37.7; 54.7)	35.9 (24.8; 44.4)	29.4 (25.0; 35.5)	<0.001
Mental component summary	47.1 (41.5; 54.5)	49.8 (35.4; 56.3)	30.1 (24.5; 34.9)	0.01

Data are means (± SD), medians (IQR); comparisons across CKD-stages using Kruskal-Wallis test; *abbreviations:* *eGFR* estimated glomerular filtration rate according to the modification of diet in renal disease formula, *RRT* renal replacement therapy (dialysis, or kidney transplantation [KTx]).

### Determinants of health related quality of life

Similar to the described findings, impaired kidney function, particularly the need of RRT, was associated with markedly reduced HRQoL across all scales in univariate linear regression analysis (Tables 3a and b). Furthermore, male gender, pain and pain-therapy were significantly and meaningfully associated with lower scores on the SF-36 scales, except general and mental health. Age and a history of vascular events, neither minor nor major events, were not found to be related to HRQoL. This is shown in detail in Additional file 1. In multivariate modeling, impaired kidney function and pain emerged as the main factors associated with lower scores on the SF 36, in particular on dimensions of physical aspects of life, such as physical functioning, role physical, bodily pain, general health and the PCS, while controlling for gender, vascular event and pain-therapy. In contrast, only the need for RRT was found to be associated with reduced HRQoL in mental/social parameter dimensions, when adjusting for the described variables. By including the respective interaction term, we tested whether the relationship of pain and HRQoL was altered by pain-therapy. Being on pain-therapy, but still suffering from pain was related to even lower scores on the role physical-scale (p = 0.001), while this finding approached significance in bodily pain (p = 0.06), role emotional (p = 0.08) and the PCS (p = 0.07). The variables included in the multivariate models explained a median of 35% (min.-max; 10-52%) of the variability in the respective SF-36 scale.

**Table 3:** **Risk factors for HRQoL, linear regression model**

	Physical functioning		Role physical		Bodily pain		General health	
	Univariate	Multivariate	Univariate	Multivariate	Univariate	Multivariate	Univariate	Multivariate
**Age (year)**	−0.1 (−0.5; 0.4)	--	−0.2 (−1.0; 0.6)	--	0.3 (−0.3; 0.8)	--	−0.03 (−0.4; 0.4)	--
**Gender (male)**	**−14.8 (−25.6; −4.0)**	−1.7 (−12.1; 8.6)	**−33.2 (−51.6; −14.9)**	−17.1 (−37.1; 2.9)	**−18.1 (−31.9; −4.3)**	0.1 (−13.1; 13.2)	−21.6 (−30.8; −12.4)	**−12.4 (−22.7; −2.1)**
**Diagnosis (month)** ^**a**^	−2.4 (−6.8; 1.9)	--	0.1 (−7.6; 7.8)	--	0.3 (−5.3; 5.9)	--	−3.3 (−7.2; 0.73)	--
**Kidney function** ^**b**^								
<60 vs. >60 ml/min/1.73 m^2^	**−25.6 (−39.8; −11.4)**	**−16.3 (−29.9; −2.7)**	−25.1 (−52.6; 2.5)	−14.8 (−41.1; 11.4)	−14.0 (−33.7; 5.7)	−0.9 (−18.1; 16.3)	−15.8 (−29.4; −2.1)	−9.3 (−22.7; 4.1)
RRT vs. >60 ml/min/1.73 m^2^	**−31.9 (−46.7; −17.0)**	**−22.7 (−39.6; −5.9)**	**−48.2 (−78.6; −17.8)**	**−34.1 (−66.6; −1.5)**	**−36.3 (−57.0; −15.6)**	**−32.5 (−53.8; −11.2)**	−32.3 (−46.7; −18.0)	**−28.8 (−45.4; −12.2)**
**Vascular event**								
Minor^c^	−4.4 (−17.7; 8.9)	4.3 (−7.2; 15.8)	−9,5 (−33.7; 14.6)	−10.5 (−32.7; 11.7)	−9.1 (−26.3; 8.0)	−2.6 (−17.1; 11.9)	−10.3 (−23.0; 2.5)	−6.6 (−18.4; 5.1)
Major^d^	10.2 (−18.0; 38.3)	18.4 (−3.7; 40.6)	2.4 (−48.6; 53.4)	19.1 (−23.6; 61.8)	18.3 (−18.0; 54.7)	24.6 (−3.4; 52.5)	−12.8 (−39.1; 13.5)	−5.0 (−26.7; 16.8)
**Pain**	**−23.3 (−23.3; −13.3)**	**−14.4 (−24.4; −4.4)**	**−37.7 (−56.3; −19.1)**	**−19.9 (−39.2; −0.6)**	**−37.2 (−49.6; −24.8)**	**−28.9 (−41.6; −16.3)**	−22.2 (−32.4; −12.2)	**−12.5 (−22.6; −2.5)**
**Pain-therapy**	**−20.1 (−32.1; −8.1)**	−10.4 (−22.0; 1.1)	**−38.8 (−58.6; −18.0)**	−16.4 (−38.6; 5.8)	**−26.0 (−40.9; −11.1)**	−10.3 (−24.8; 4.3)	−19.2 (−30.3; −8.1)	−2.8 (−14.2; 8.7)
	**Vitality**		**Social functioning**		**Role emotional**		**Mental health**	
	**Univariate**	**Multivariate**	**Univariate**	**Multivariate**	**Univariate**	**Multivariate**	**Univariate**	**Multivariate**
**Age (year)**	−0.1 (−0.5; 0.3)	--	−0.1 (−0.6; 0.4)	--	−0.5 (−1.2; 0.2)	--	−0.1 (−0.4; 0.3)	--
**Gender (male)**	**−12.1 (−22.3; −1.9)**	0.5 (−10.6; 11.6)	**−18.2 (−29.8; −6.5)**	−4.2 (−15.6; 7.2)	**−22.8 (−40.1; −5.5)**	−8.6 (−26.9; 9.8)	−7.7 (−16.1; 0.8)	−0.9 (−10.3; 8.5)
**Diagnosis (month)** ^**a**^	−1.1 (−5.2; 3.1)	--	0.3 (−4.5; 5.1)	--	−4.6 (−11.6; 2.3)	--	0.7 (−2.7; 4.0 )	--
**Kidney function** ^**b**^								
<60 vs. >60 ml/min/1.73 m^2^	−6.7 (−21.1; 7.7)	−1.5 (−15.9; 13.0)	−3.9 (−19.6; 12.1)	3.0 (−11.9; 18.0)	−15.6 (−40.1; 8.8)	−6.6 (−30.4; 17.2)	−2.0 (−14.0; 10.1)	1.9 (−10.4; 14.2)
RRT vs. >60 ml/min/1.73 m^2^	**−25.3 (−40.4; −10.2)**	**−24.5 (−42.4; −6.6)**	**−41.5 (−58.3; −24.7)**	**−46.2 (−64.7; −27.6)**	**−45.7 (−71.3; −20.0)**	**−45.0 (−74.6; −15.5)**	**−17.4 (−30.0; −4.8)**	**−15.8 (−31.0; −0.6)**
**Vascular event**								
Minor^c^	−1.0 (−13.8; 11.9)	−0.7 (−13.4; 12.0)	−0.66 (−15.5; 14.2)	0.5 (−12.1; 13.2)	−4.1 (−25.6; 17.5)	0.4 (−19.7; 20.5)	−3.0 (−13.6; 7.6)	−1.5 (−12.3; 9.3)
Major^d^	−13.0 (−39.5; 13.5)	−14.1 (−37.7; 9.4)	10.8 (−20.7; 42.3)	11.2 (−13.1; 35.6)	5.0 (−40.7; 50.6)	7.2 (−31.6; 46.0)	−5.1 (−26.9; 16.7)	−5.4 (−25.4; 14.6)
**Pain**	**−19.8 (−29.7; −10.0)**	**−14.8 (−25.7; −3.9)**	**−20.0 (−31.8; −8.2)**	−9.3 (−20.3; 1.8)	**−20.8 (−38.1; −3.5)**	−5.1 (−22.8; 12.6)	−8.1 (−16.7; 0.5)	−1.6 (−10.9; 7.6)
**Pain-therapy**	−10.2 (−21.9; 1.4)	−0.6 (−13.0; 11.7)	**−14.6 (−28.1; −1.1)**	−7.6 (−20.3; 5.1)	−14.6 (−34.6; 5.3)	−8.1 (−28.4; 12.2)	−6.6 (−16.0; 2.8)	−4.8 (−15.3; 5.6)
		**Physical component summary**				**Mental component summary**		
		**Univariate**		**Multivariate**		**Univariate**		**Multivariate**
**Age (year)**		0.03 (−0.2; 0.2)		--		−0.1 (−0.39; 0.1)		--
**Male gender**		**−8.9 (−13.8; −4.0)**		−2.4 (−7.3; 2.5)		**−4.9 (−9.8; −0.02)**		−0.2 (−6.2; 4.5)
**Diagnosis (month)** ^**a**^		−0.6 (−2.7; 1.4)		--		−0.3 (−2.2; 1.7)		--
**Kidney function** ^**b**^								
<60 vs. >60 ml/min/1.73 m^2^		**−10.4 (−17.3; −3.4)**		−6.2 (−12.5; 0.1)		1.1 (−5.7; 7.9)		2.8 (3.5; −4.1)
RRT vs. >60 ml/min/1.73 m^2^		**−16.0 (−23.7; −8,4)**		**−11.8 (−19.6; −4.0)**		**−14.3 (−21.8; −6.8)**		**−13.3 (−21.8; −4.7)**
**Vascular event**								
Minor^c^		−2.9 (−9.5; 3.7)		−0.1 (−5.5; 5.4)		−0.6 (−6.8; 5.6)		−1.1 (−7.1; 4.9)
Major^d^		3.3 (−10.3; 16.8)		8.2 (−2.0; 18.4)		−1.1 (−13.9; 11.7)		−3.4 (−14.6; 7.8)
**Pain**		**−14.3 (−18.9; −9.7)**		**−9.1 (−13.8; −4.3)**		−4.1 (−9.0; 0.9)		−1.1 (−6.3; 4.1)
**Pain-therapy**		−12.2 (−17.7; 6.8)		**−5.5 (−10.9; −0.1)**		−1.8 (−7.4; 3.7)		−0.4 (−6.3; 5.6)

Data are beta-coefficients with respective 95% confidence interval (CI), significant values (p < 0.05) bolded.

^a^Diagnosis Fabry disease, logarithmic form.

^b^Patient with kidney transplantation classified as RRT.

^c^History of transient ischemic attack (TIA), angina pectoris, significant cardiac procedure without coronary stent, arrhythmia.

^d^History of stroke, myocardial infarction, coronary stent, coronary bypass surgery, ICD-implantation.

We tested the robustness of our results in a first step by including only four explanatory variables in the multivariate linear regression model. Excluding vascular event, which represented the least significant variable, did not result in significant changes of our findings, neither on the magnitude of the direction, nor on the significance levels or the respective variables. Similarly, by including three additional patients in whom the SF-36 was completed within nine months prior or following the study visit, no significant changes in the estimates of the variables in the multivariate model were found; however, the 95% CI became narrower, resulting in pain-therapy being significant on the p < 0.05 level in the physical functioning scale. Moreover, when we analyzed the two KTx-patients in the respective eGFR category, i.e., excluding them from the RRT category, we did not detect major differences in the findings, except in the variable describing kidney function. Generally, the associations of RRT with the respective scales strengthened including narrower 95% CI in physical aspects, such as physical functioning, role physical and emotional, and the PCS. No changes were observed in mental parameters. Details are shown in Additional file 1. Finally, treating CV-events in a single category did also not alter the results: neither became the variable CV-event significant, nor did the associations of the remaining determinants in the model substantially change. Additional file 1 shows this in more detail.

## Discussion

Chronic kidney disease was generally associated with reduced HRQoL, especially mental health, as represented by social functioning, role emotional, and the MCS. In contrast, physical aspects, such as physical functioning, role physical, bodily pain, and the PCS, were affected even in patients with moderately impaired kidney function. Impaired kidney function, particularly the need of RRT, was associated with markedly reduced HRQoL across all scales in univariate linear regression analysis.

A number of studies investigated Quality of life in patients with Fabry disease. Most studies constrain to comparisons with healthy controls which indicate major impairments in affected patients [8, 9, 16, 17] and the effects of enzyme replacement therapy on HRQoL. If SF-36 questionnaires were used, the main affected parameter was ‘general health’ [9]. Moreover, psychiatric diseases and related affections of HRQoL are frequently evident in Fabry patients [18]. Data from the Fabry outcome survey showed reduced values for EQ-5D in FD compared to healthy controls and other diseases like lung cancer, kidney and liver transplantation [19]. Improvements of HRQoL were also reported after 12 months of ERT. [17, 20–22], although it is not completely understood, how treatment directly improves quality of life. None of the studies reported the HRQoL according to specific organ dysfunction. Chronic neuropathic pain is still regarded as one of the most difficult aspects of Fabry's disease for patients. Its effect on HRQoL is still regarded larger than cardiac and renal disease [23]. The long-term complications in kidney, heart and brain lead to renal failure, cardiomyopathy, and strokes in adults, respectively and are all well known to impact on patients’ wellbeing and social function in various magnitude. However, in our study, the history of minor of major vascular events including stroke or cardiac complications was not significantly associated with HRQoL.

We found that chronic kidney disease, although impaired kidney function and its progression is deemed to be apparently asymptomatic, was an important determinant of the patients’ wellbeing which is a crucial factor for reduced HRQoL in physical and mental and social aspects of life. Among the SF-36, reduced kidney function was connected to reduced values in all eight items, mainly affecting physical role (univariate −48.2 (−78.6; −17,8)) and social functioning (univariate −41.5 (−58.3; 24.7)) which is considered as clinically meaningful [24]. While in particular the need of RRT was associated with dramatic impairments of all aspects of HRQoL, even non-dialysis-dependent CKD was related to lower scores in physical dimensions. These findings were evident, however somewhat attenuated, after controlling for clinical variables including male gender.

Major therapeutic components of Fabry disease patients focus on pain medication and mental coping, including modifications of daily life activities and behavior. Suffering from pain naturally can be seen as causal for reductionsin social and physical functioning. In our analyses it was interesting that being on specific-medication, but still suffering from pain was related to even lower scores on the role physical-scale, while this finding approached significance in bodily pain, role emotional and the PCS.This might either be caused by a higher level of pain which is pledged to analgetics to reach a bearable level but is still affecting quality of life, or might be connected to the additional negative effect of destitution by ineffective pain medication.

It has been suggested by various studies [8, 9] that a history of cardiovascular disease impacts on HRQoL in Fabry patients. We categorized cardiovascular events into minor and major events and found that neither was significantly associated with HRQoL in univariate or multivariate analyses, respectively. Besides that our data show the importance of kidney function which was not addressed in previous studies.

In contrast, in a large analysis of Fabry patients in Canada [25], patients had more neurological events, more cardiac events and more often pain to an extent that made them candidate for enzyme therapy. The fact that data of our cohort were analyzed at the patients’ first visit in our centre, and were also slightly younger (40 ± 13 yrs) than the cohort by Sirrs et al. (48 ± 16 yrs), might be the reason for out cohort reporting a history of CV-events less frequently. However, the absolute frequency of vascular events of about 10-20% and frequency of pain in 70% is fairly common in Fabry disease (FOS - Fabry outcome survey) [26].

Our findings indicate that kidney disease in FD should be consideredwhen aiming for improvements of HRQoL. Aside from enzyme replacement therapy, optimal treatment of kidney disease aiming to slow the progression of CKD and an effective pain therapy may help to improve HRQoL in Fabry patients.

### Limitations

We are aware of several limitations of our findings. As in any observational study, no relationship can be determined sufficiently, i.e., the direction of an association in cross-sectional analyses cannot be explored. It would also be desirable to investigate the changes in HRQoL in FD patients and how these may be associated with changes in renal function, optimized pain therapy, the occurrence of vascular events, but also with the potential effects of ERT. Moreover, the differentiation of pain severity according to receiving or not receiving therapy for neurogenic pain is diffuse, because it may depend on differences in perception of pain but also on differences in patient care. Besides that, an influence of pain peaks, which are not detected by the use of the VAS at one single point of time, on HRQoL is not excluded.

## Conclusions

Cardiovascular events and neuropathic pain have been considered as solely being responsible for reduced quality of life, depression as well as reduced social functioning. Our study highlights chronic kidney disease, in particular after initiation of RRT, but even early in disease progression, as a strong determinant of reduced HRQoL in Fabry’s disease.

## Electronic supplementary material

Additional file 1: **Supplementary materials.** Description S1) HRQoL data of patients with a history of major CV-events. S2) Sensitivity analysis: KTx patients considered in the respective CKD category, according to eGFR; determinants of HRQoL, displayed are β- coefficients (95% CI). S3) Sensitivity analysis: CV-events treated as a single category; determinants of HRQoL, displayed are β-coefficients (95% CI). (DOCX 32 KB)
