# Supplementary material for: Kidney function as an underestimated factor for reduced health related quality of life in patients with Fabry disease
Source: BMC Nephrol. 2014 Nov 29;15:188. doi: 10.1186/1471-2369-15-188 (PMC4280765; doi:10.1186/1471-2369-15-188)
Supplement: Supplementary file 1 — Additional file 1: Supplementary materials. Description S1) HRQoL data of patients with a history of major CV-events. S2) Sensitivity analysis: KTx patients considered in the respective CKD category, according to eGFR; determinants of HRQoL, displayed are β- coefficients (95% CI). S3) Sensitivity analysis: CV-events treated as a single category; determinants of HRQoL, displayed are β-coefficients (95% CI). (DOCX 32 KB) [file 12882_2014_888_MOESM1_ESM.docx]

**Supplementary materials**

Wagner M, Krämer J *et al.* Kidney function as an underestimated factor for reduced health related quality of life in patients with Fabry disease

**S1) HRQoL data of patients with a history of major CV-events**

| **ID** | **age** | **gender** | **Diagnosis Fabry [yrs]** | **CKD** | **Physical**  **functioning** | **Role**  **physical** | **Bodily**  **pain** | **General**  **health** | **Vitality** | **Social**  **functioning** | **Role**  **emotional** | **Mental**  **health** | **Physical**  **component score** | **Mental**  **component score** |
| --- | --- | --- | --- | --- | --- | --- | --- | --- | --- | --- | --- | --- | --- | --- |
| 1 | 37 | male | 4.1 | No | 75 | 50 | 100 | 52 | 40 | 87.5 | 100 | 40 | 48.7 | 42.4 |
| 2 | 37 | male | 13.2 | No | 90 | 50 | 80 | 47 | 50 | 87.5 | 33.3 | 60 | 49.7 | 39.5 |
| 3 | 55 | male | 7.6 | Yes | 85 | 100 | 64 | 35 | 10 | 75 | 100 | 76 | 44.4 | 49.4 |
| 4 | 63 | male | 10.9 | No |  |  |  |  |  |  |  |  |  |  |

**S2) Sensitivity analysis: KTx patients considered in the respective CKD category, according to eGFR; determinants of HRQoL, displayed are β-coefficients (95% CI)**

|  | **Physical functioning** | **Role physical** | **Bodily pain** | **General health** | **Vitality** | **Social functioning** | **Role emotional** | **Mental health** | **Physical component**  **score** | **Mental component**  **score** |
| --- | --- | --- | --- | --- | --- | --- | --- | --- | --- | --- |
| **gender (male)** | -1.4  (-11.7; 8.9) | -17.8  (-37.8; 2.2) | 1.0  (-11.7; 13.7) | **-12.6**  **(-22.9; -2.4)** | 0.9  (-12.2; 10.4) | -5.1  (-16.8; 6.6) | -10.6  (-29.2; 8.1) | -0.7  (-10.0; 8.6) | -2.2  (-7.1; 2.6) | -1.4  (-6.9; 4.1) |
| **Chronic kidney disease** |  |  |  |  |  |  |  |  |  |  |
| *<60 vs. >60  ml/min/1.73m²* | **-15.8**  **(-28.9; -2.6)** | -17.1  (-42.7; 8.3) | -0.4  (-15.8; 16.6) | -10.4  (-23.4; 2.6) | -5.5  (-19.8; 8.8) | -0.7  (-15.7; 14.2) | -12.4  (-35.9; 11.1) | 1.9  (-10.0; 13.7) | -5.9  (-12.0; 0.1) | 1.0  (-5.8; 7.9) |
| *RRT vs. >60*  *ml/min/1.73m²* | **-24.9**  **(-42.8; -7.0)** | -32.1  (-66.8; 2.6) | **-40.4**  **(-62.5; -18.3)** | **-29.6**  **(-47.3; -12.0)** | **-19.7**  **(-39.2; -0.3)** | **-46.1**  **(-66.4; -25.8)** | **-38.7**  **(-70.7; -6.6)** | **-18.6**  **(-34.6; -2.5)** | **-13.2**  **(-21.4; -5.0)** | **-12.1**  **(-21.4; -2.7)** |
| **vascular event** |  |  |  |  |  |  |  |  |  |  |
| *Minor* | 4.5  (-7.0; 15.9) | -9.7  (-31.9; 12.5) | -1.5  (-15.6; 12.6) | -5.9  (-17.6; 5.8) | -0.4  (-12.5; 13.3) | 2.4  (-10.6; 15.4) | 1.9  (-18.6; 22.4) | -0.8  (-11.5; 9.8) | 0.2  (-5.3; 5.6) | -0.4  (-6.6; 5.8) |
| *Major* | 18.2  (-3.7; 40.2) | 20.8  (-21.8; 63.6) | 24.4  (-2.6; 51.5) | -4.0  (-25.7; 17.7) | -11.4  (-35.3; 12.5) | 14.2  (-10.7; 39.1) | 11.2  (-28.1; 50.5) | -5.0  (-24.7; 14.7) | 8.1  (-1.9; 18.2) | -2.1  (-13.5; 9.4) |
| **pain_mod** | **-14.7**  **(-24.7; -4.6)** | **-19.7**  **(-39.1; -0.3)** | **-29.9**  **(-42.2; -17.5)** | **-12.6**  **(-22.7; -2.5)** | **-14.2**  **(-25.3; -3.1)** | -9.3  (-20.7; 2.1) | -4.5  (-22.5; 13.6) | -2.0  (-11.1; 7.2) | **-9.2**  **(-14.0; -4.5)** | -0.9  (-6.3; 4.4) |
| **pain_therapy** | -10.7  (-22.2; 0.8) | -17.0  (-39.2; 5.3) | -11.5  (-25.7; 2.7) | -3.4  (-15.0; 8.0) | -1.3  (-14.0; 11.3) | -9.2  (-22.2; 3.9) | -9.1  (-29.8; 11.6) | -5.4  (-15.9; 5.0) | **-5.8**  **(-11.1; -0.04)** | -0.9  (-7.0; 5.2) |

**S3) Sensitivity analysis: CV-events treated as a single category; determinants of HRQoL, displayed are β-coefficients (95% CI)**

|  | **Physical**  **functioning** | **Role**  **physical** | **Bodily**  **pain** | **General**  **health** | **Vitality** | **Social**  **functioning** | **Role**  **emotional** | **Mental**  **health** | **Physical**  **component score** | **Mental**  **component score** |
| --- | --- | --- | --- | --- | --- | --- | --- | --- | --- | --- |
| **gender (male)** | 0.02  (-10.0; 10.1) | -13.4  (-32.8; 6.0) | 3.4  (-9.5; 16.2) | **-12.2**  **(-22.1; -2.3)** | -1.1  (-11.8; 9.7) | -2.9  (-13.9; 8.1) | -7.7  (-25.3; 9.9) | -1.4  (-10.5; 7.7) | -1.4  (-6.2; 3.4) | -1.1  (-6.2; 4.0) |
| **Chronic kidney disease** |  |  |  |  |  |  |  |  |  |  |
| *<60 vs. >60  ml/min/1.73m²* | **-16.1**  **(-29.8; -2.3)** | -14.3  (-40.8; 12.2) | -0.4  (-18.0; 17.1) | -9.3  (-22.7; 4.1) | -1.7  (-16.3; 12.9) | 3.2  (-11.8; 18.3) | -6.4  (-30.3; 17.4) | 1.8  (-10.4; 14.1) | -6.1  (-12.4; 0.3) | 2.8  (-4.1; 9.7) |
| *RRT vs. >60*  *ml/min/1.73m²* | **-24.2**  **(-41.1; -7.4)** | **-37.2**  **(-69.7; -4.6)** | **-35.3**  **(-56.8; -13.8)** | **-29.0**  **(-45.4; -12.6)** | **-23.1**  **(-41.0; -5.3)** | **-47.3**  **(-65.7; -28.9)** | **-45.8**  **(-75.1; -16.5)** | **-15.4**  **(-30.5; -0.4)** | **-12.6**  **(-20.4; -4.8)** | **-13.0**  **(-21.5; -4.6)** |
| **vascular event, any** | 6.8  (-4.0; 17.6) | -5.4  (-26.0; 15.7 | 2.3  (-11.5; 16.1) | -6.3  (-17.2; 4.5) | -3.2  (-15.1; 8.6) | 2.5  (-9.4; 14.2) | 1.6  (-17.1; 20.4) | -2.2  (-12.2; 7.7) | 1.5  (-3.7; 6.7) | -1.5  (-7.1; 4.1) |
| **pain_mod** | **-15.2**  **(-25.2; -5.2)** | **-21.5**  **(-40.9; -2.2)** | **-30.4**  **(-43.3; -17.6)** | **-12.6**  **(-22.6; -2.6)** | **-14.2**  **(-25.0; -3.3)** | -9.9  (-20.8; 1.1) | -5.5  (-23.0; 12.1) | -1.5  (-10.6; 7.7) | **-9.4**  **(-14.3; -4.6)** | -1.0  (-6.2; 4.2) |
| **pain_therapy** | -10.2  (-21.9; 1.4) | -16.0  (-38.4; 6.5) | -9.9  (-24.8; 4.9) | -2.8  (-14.2; 8.7) | -0.7  (-13.2; 11.8) | -7.5  (-20.2; 5.2) | -8.0  (-28.3; 12.3) | -4.8  (-15.3; 5.7) | **-5.5**  **(-11.0; 0.00)** | -0.4  (-6.3; 5.6) |
